# Supplementary material for: Multi‐dataset identification of innovative feature genes and molecular mechanisms in keratoconus
Source: J Cell Mol Med. 2024 Sep 19;28(18):e70079. doi: 10.1111/jcmm.70079 (PMC11412914; doi:10.1111/jcmm.70079)
Supplement: Supplementary file 2 — Material S2. [file JCMM-28-e70079-s002.zip › 7.GSEA analysis/5.RNF217-AS1/KEGG_UBIQUITIN_MEDIATED_PROTEOLYSIS.html]

Details for gene set KEGG\_UBIQUITIN\_MEDIATED\_PROTEOLYSIS[GSEA]

|  || Dataset | RNF217-AS1.group.cls#h\_versus\_l |
| Phenotype | group.cls#h\_versus\_l |
| Upregulated in class | l |
| GeneSet | KEGG\_UBIQUITIN\_MEDIATED\_PROTEOLYSIS |
| Enrichment Score (ES) | -0.2235409 |
| Normalized Enrichment Score (NES) | -1.2905765 |
| Nominal p-value | 0.17255718 |
| FDR q-value | 0.4010203 |
| FWER p-Value | 0.961 |
Table: GSEA Results Summary

  

Fig 1: Enrichment plot: KEGG\_UBIQUITIN\_MEDIATED\_PROTEOLYSIS      
 Profile of the Running ES Score & Positions of GeneSet Members on the Rank Ordered List

  

| SYMBOL | TITLE | RANK IN GENE LIST | RANK METRIC SCORE | RUNNING ES | CORE ENRICHMENT || 1 | MID1 | na | 2307 | 0.327 | -0.0322 | No |
| 2 | HERC4 | na | 7479 | 0.141 | -0.1669 | No |
| 3 | CBL | na | 7653 | 0.136 | -0.1575 | No |
| 4 | PIAS1 | na | 7853 | 0.131 | -0.1495 | No |
| 5 | CBLC | na | 8003 | 0.128 | -0.1403 | No |
| 6 | TRIP12 | na | 8400 | 0.119 | -0.1393 | No |
| 7 | UBE2O | na | 8473 | 0.117 | -0.1291 | No |
| 8 | SIAH1 | na | 8692 | 0.112 | -0.1236 | No |
| 9 | CBLB | na | 8913 | 0.107 | -0.1187 | No |
| 10 | UBE2I | na | 9244 | 0.100 | -0.1177 | No |
| 11 | UBR5 | na | 9646 | 0.092 | -0.1195 | No |
| 12 | HERC2 | na | 9806 | 0.090 | -0.1147 | No |
| 13 | UBE4B | na | 9828 | 0.089 | -0.1059 | No |
| 14 | UBA3 | na | 9984 | 0.087 | -0.1013 | No |
| 15 | UBE2QL1 | na | 10056 | 0.085 | -0.0943 | No |
| 16 | UBE2H | na | 10254 | 0.082 | -0.0914 | No |
| 17 | HUWE1 | na | 10368 | 0.080 | -0.0862 | No |
| 18 | UBE2D1 | na | 10791 | 0.073 | -0.0907 | No |
| 19 | UBE2U | na | 10854 | 0.072 | -0.0848 | No |
| 20 | NEDD4L | na | 11023 | 0.070 | -0.0824 | No |
| 21 | UBE2E1 | na | 11195 | 0.067 | -0.0802 | No |
| 22 | UBE2W | na | 11219 | 0.067 | -0.0738 | No |
| 23 | UBE3C | na | 11444 | 0.064 | -0.0736 | No |
| 24 | PIAS4 | na | 11448 | 0.064 | -0.0670 | No |
| 25 | UBE2G1 | na | 11990 | 0.055 | -0.0768 | No |
| 26 | FANCL | na | 12374 | 0.050 | -0.0826 | No |
| 27 | UBE2NL | na | 12472 | 0.049 | -0.0802 | No |
| 28 | ANAPC1 | na | 12601 | 0.047 | -0.0789 | No |
| 29 | SMURF1 | na | 12661 | 0.047 | -0.0757 | No |
| 30 | VHL | na | 13248 | 0.039 | -0.0885 | No |
| 31 | CDC34 | na | 13655 | 0.034 | -0.0966 | No |
| 32 | UBE3A | na | 13725 | 0.034 | -0.0951 | No |
| 33 | MGRN1 | na | 13825 | 0.032 | -0.0945 | No |
| 34 | XIAP | na | 13938 | 0.031 | -0.0945 | No |
| 35 | WWP2 | na | 14267 | 0.027 | -0.1011 | No |
| 36 | MDM2 | na | 14406 | 0.026 | -0.1023 | No |
| 37 | UBE2L3 | na | 14433 | 0.025 | -0.1004 | No |
| 38 | UBE2D3 | na | 14741 | 0.022 | -0.1070 | No |
| 39 | UBE2F | na | 15006 | 0.019 | -0.1126 | No |
| 40 | ANAPC5 | na | 15025 | 0.019 | -0.1112 | No |
| 41 | NEDD4 | na | 15224 | 0.017 | -0.1151 | No |
| 42 | FZR1 | na | 15874 | 0.010 | -0.1328 | No |
| 43 | PRPF19 | na | 16123 | 0.008 | -0.1392 | No |
| 44 | CDC23 | na | 16140 | 0.007 | -0.1389 | No |
| 45 | UBE2Z | na | 16172 | 0.007 | -0.1390 | No |
| 46 | RBX1 | na | 16317 | 0.006 | -0.1426 | No |
| 47 | FBXW11 | na | 16324 | 0.006 | -0.1422 | No |
| 48 | NHLRC1 | na | 16379 | 0.005 | -0.1432 | No |
| 49 | SKP1 | na | 16603 | 0.003 | -0.1494 | No |
| 50 | ANAPC7 | na | 16717 | 0.001 | -0.1525 | No |
| 51 | CUL4B | na | 16841 | 0.000 | -0.1560 | No |
| 52 | UBE2A | na | 16882 | -0.000 | -0.1571 | No |
| 53 | UBE2R2 | na | 17364 | -0.005 | -0.1705 | No |
| 54 | KEAP1 | na | 17566 | -0.007 | -0.1756 | No |
| 55 | WWP1 | na | 17624 | -0.007 | -0.1764 | No |
| 56 | UBE2D2 | na | 17734 | -0.008 | -0.1787 | No |
| 57 | BIRC2 | na | 17820 | -0.009 | -0.1802 | No |
| 58 | UBA6 | na | 17898 | -0.010 | -0.1814 | No |
| 59 | ANAPC11 | na | 18123 | -0.012 | -0.1866 | No |
| 60 | CDC20 | na | 18128 | -0.012 | -0.1855 | No |
| 61 | AIRE | na | 18315 | -0.014 | -0.1894 | No |
| 62 | CDC27 | na | 18332 | -0.014 | -0.1885 | No |
| 63 | ITCH | na | 18557 | -0.016 | -0.1932 | No |
| 64 | PML | na | 18765 | -0.018 | -0.1973 | No |
| 65 | ANAPC13 | na | 18874 | -0.019 | -0.1984 | No |
| 66 | SAE1 | na | 19001 | -0.020 | -0.2000 | No |
| 67 | UBOX5 | na | 19247 | -0.023 | -0.2047 | No |
| 68 | BTRC | na | 19413 | -0.024 | -0.2069 | No |
| 69 | HERC1 | na | 19542 | -0.026 | -0.2079 | No |
| 70 | PPIL2 | na | 19553 | -0.026 | -0.2055 | No |
| 71 | CUL4A | na | 19597 | -0.026 | -0.2039 | No |
| 72 | PIAS2 | na | 19967 | -0.030 | -0.2115 | No |
| 73 | TRAF6 | na | 20014 | -0.030 | -0.2096 | No |
| 74 | UBE2S | na | 20128 | -0.031 | -0.2096 | No |
| 75 | BIRC3 | na | 20203 | -0.032 | -0.2084 | No |
| 76 | UBE2Q2 | na | 20291 | -0.033 | -0.2074 | No |
| 77 | UBE2G2 | na | 20337 | -0.033 | -0.2052 | No |
| 78 | CUL5 | na | 20366 | -0.034 | -0.2025 | No |
| 79 | UBE2K | na | 20383 | -0.034 | -0.1994 | No |
| 80 | STUB1 | na | 20394 | -0.034 | -0.1961 | No |
| 81 | CDC16 | na | 20405 | -0.034 | -0.1929 | No |
| 82 | ANAPC4 | na | 20735 | -0.037 | -0.1985 | No |
| 83 | PIAS3 | na | 20986 | -0.040 | -0.2015 | No |
| 84 | UBA1 | na | 21023 | -0.040 | -0.1983 | No |
| 85 | UBE4A | na | 21339 | -0.043 | -0.2028 | No |
| 86 | UBE2J1 | na | 21352 | -0.044 | -0.1986 | No |
| 87 | ANAPC10 | na | 21518 | -0.045 | -0.1986 | No |
| 88 | BIRC6 | na | 21649 | -0.047 | -0.1974 | No |
| 89 | UBE2Q1 | na | 21706 | -0.047 | -0.1940 | No |
| 90 | CUL1 | na | 21972 | -0.050 | -0.1964 | No |
| 91 | UBE2D4 | na | 22282 | -0.054 | -0.1996 | No |
| 92 | DDB2 | na | 22664 | -0.058 | -0.2046 | No |
| 93 | UBE2E2 | na | 23322 | -0.066 | -0.2166 | Yes |
| 94 | UBA2 | na | 23334 | -0.066 | -0.2100 | Yes |
| 95 | FBXO2 | na | 23672 | -0.071 | -0.2123 | Yes |
| 96 | UBE2M | na | 23693 | -0.071 | -0.2054 | Yes |
| 97 | KLHL9 | na | 23797 | -0.072 | -0.2008 | Yes |
| 98 | CUL2 | na | 23798 | -0.072 | -0.1932 | Yes |
| 99 | CUL3 | na | 23825 | -0.073 | -0.1863 | Yes |
| 100 | RNF7 | na | 23903 | -0.074 | -0.1807 | Yes |
| 101 | SYVN1 | na | 23923 | -0.074 | -0.1735 | Yes |
| 102 | FBXW8 | na | 23990 | -0.075 | -0.1675 | Yes |
| 103 | UBE2L6 | na | 24379 | -0.081 | -0.1702 | Yes |
| 104 | TRIM37 | na | 24455 | -0.082 | -0.1638 | Yes |
| 105 | UBE3B | na | 24476 | -0.082 | -0.1557 | Yes |
| 106 | CDC26 | na | 24588 | -0.084 | -0.1501 | Yes |
| 107 | MAP3K1 | na | 24706 | -0.086 | -0.1444 | Yes |
| 108 | UBE2B | na | 24731 | -0.086 | -0.1360 | Yes |
| 109 | FBXW7 | na | 24820 | -0.087 | -0.1293 | Yes |
| 110 | ERCC8 | na | 25003 | -0.090 | -0.1251 | Yes |
| 111 | FBXO4 | na | 25043 | -0.091 | -0.1166 | Yes |
| 112 | UBE2J2 | na | 25127 | -0.092 | -0.1093 | Yes |
| 113 | DET1 | na | 25551 | -0.099 | -0.1111 | Yes |
| 114 | UBE2C | na | 26579 | -0.118 | -0.1284 | Yes |
| 115 | SKP2 | na | 27110 | -0.128 | -0.1302 | Yes |
| 116 | RHOBTB2 | na | 27411 | -0.134 | -0.1247 | Yes |
| 117 | ANAPC2 | na | 27696 | -0.141 | -0.1181 | Yes |
| 118 | DDB1 | na | 27810 | -0.144 | -0.1062 | Yes |
| 119 | CUL7 | na | 27962 | -0.148 | -0.0950 | Yes |
| 120 | UBE2E3 | na | 28006 | -0.149 | -0.0806 | Yes |
| 121 | TRIM32 | na | 28553 | -0.164 | -0.0792 | Yes |
| 122 | UBA7 | na | 28686 | -0.168 | -0.0653 | Yes |
| 123 | SMURF2 | na | 29259 | -0.184 | -0.0625 | Yes |
| 124 | UBE2N | na | 29871 | -0.202 | -0.0589 | Yes |
| 125 | RCHY1 | na | 29948 | -0.204 | -0.0396 | Yes |
| 126 | SOCS1 | na | 30781 | -0.234 | -0.0390 | Yes |
| 127 | BRCA1 | na | 31064 | -0.245 | -0.0214 | Yes |
| 128 | KLHL13 | na | 33102 | -0.368 | -0.0415 | Yes |
| 129 | HERC3 | na | 33124 | -0.370 | -0.0032 | Yes |
| 130 | SOCS3 | na | 33937 | -0.470 | 0.0229 | Yes |
Table: GSEA details [plain text format]

  

Fig 2: KEGG\_UBIQUITIN\_MEDIATED\_PROTEOLYSIS      
 Blue-Pink O' Gram in the Space of the Analyzed GeneSet

  

Fig 3: KEGG\_UBIQUITIN\_MEDIATED\_PROTEOLYSIS: Random ES distribution      
 Gene set null distribution of ES for **KEGG\_UBIQUITIN\_MEDIATED\_PROTEOLYSIS**
